# Reliability and validity of the Japanese version of the Mental Health Self-management Questionnaire among people with mental illness living in the community

**DOI:** 10.1186/s40359-019-0301-4

**Published:** 2019-05-22

**Authors:** Yasuko Morita, Yuki Miyamoto, Ayumi Takano, Norito Kawakami, Simon Coulombe

**Affiliations:** 1 Department of Psychiatric Nursing, Graduate School of Medicine, The University of Tokyo, 7-3-1, Hongo, Bunkyo-ku, Tokyo, 113-0033 Japan; 2 Department of Mental Health and Psychiatric Nursing, Tokyo Medical and Dental University, 1-5-45, Yushima, Bunkyo-ku, Tokyo, 113-8510 Japan; 3 Department of Mental Health, Graduate School of Medicine, The University of Tokyo, 7-3-1, Hongo, Bunkyo-ku, Tokyo, 113-0033 Japan; 4 Department of Psychology, Faculty of Science, Wilfrid Laurier University, 75 University Avenue West, Waterloo, Ontario N2L 3C5 Canada

**Keywords:** Mental health, Mental illness, Self-management, Community, Outpatient

## Abstract

**Background:**

Self-management is an important factor in maintaining and promoting mental health and recovery from mental health challenges. Thus, it is important to assess and support mental health self-management. In this study, we aimed to develop the Japanese version of the Mental Health Self-management Questionnaire (MHSQ-J), a scale to assess mental health self-management strategy, and clarify its psychometric properties among people with mental illness living in Japan.

**Methods:**

An anonymous self-administered survey including MHSQ-J was conducted for psychiatric outpatient users (*N* = 295), and 104 of the participants completed MHSQ-J again about two weeks later. Internal consistency was assessed with Cronbach’s α, and test-retest reliability was confirmed by the intraclass correlation coefficient (ICC). Construct validity was assessed based on structural validity with confirmatory factor analysis (CFA) and exploratory factor analysis (EFA), and hypotheses testing. The Self-management Skill Scale, the University of Tokyo Health Sociology version of the Sense of Coherence Scale ver1.2, the Japanese version of Self-identified Stage of Recovery Part-B, the Japanese version of the Flourishing Scale, and the Japanese version of the WHO Disability Assessment Scale 2.0 were used for hypotheses testing.

**Results:**

Data from 243 respondents were analyzed. The result of CFA, the goodness-of-fit indices showed marginal fit (AGFI = .830, CFI = .852, RMSEA = .072). EFA identified three factors (Clinical, Empowerment, and Vitality), and the results suggested that the factor structure of the Japanese version of MHSQ was similar to the original 3-factor structure. Significant correlations were found with the hypotheses testing variables related to self-management and recovery, especially on the total score, the Empowerment subscale, and the Vitality subscale. Cronbach’s α (Clinical: .65, Empowerment: .81, Vitality: .75, Total: .83) and ICC (Clinical: .75, 95% confidence interval (CI) [.62, .84], Empowerment: .81, 95% CI [.70, .88], Vitality: .62, 95% CI [.44, .75], Total: .84, 95% CI [.75, .90]) indicated good reliability.

**Conclusion:**

The results show that MHSQ-J has acceptable reliability and validity to measure the use of self-management strategies for mental health among community living people with mental illness in Japan.

**Electronic supplementary material:**

The online version of this article (10.1186/s40359-019-0301-4) contains supplementary material, which is available to authorized users.

## Background

Self-management is a subjective day-to-day approach including medical management, role management and emotional aspects of their condition, which is engaged to improve health conditions and maintain wellness [1]. This self-management starts with a person addressing their own difficulties and concerns in their daily life [1]. Self-management improves several aspects of life with chronic illness, such as symptoms, self-efficacy and Quality of Life (QOL) [2, 3].

Mental health guidelines and guidance have pointed out the importance of self-management to complement pharmacotherapy and psychotherapy in the treatment of mental illness [4–6]. The reported benefits of self-management for mental health include higher self-efficacy, a lower relapse rate, higher sense of coherence, better self-rated health, and fewer comorbidities [7, 8]. Additionally, self-management is essential to promoting “recovery” [8]. Recovery means not only improvement in clinical aspects such as a reduction of symptoms, but also personal aspects such as transformation of attitudes or ways of thinking, enabling patients to live their life more satisfactorily and with hope [9, 10].

To support the recovery of the people with mental health challenges, it is important to assess and support their self-management behavior as the outcome of their self-management. Behavior is all actions, not only those externally observable, but also inner actions like thoughts or recognitions. In the mental health area, most of the scales for measuring self-management are limited by the diagnosis or situation of use such as a psychiatric vocational rehabilitation service. Nowadays, much of the mental health welfare support services within a community are provided to users without the distinction of a diagnosis. Furthermore, a program style that doesn’t require information regarding the diagnosis is not uncommon when the aim is to facilitate recovery. Thus, a trans-diagnostic scale that measures self-management behavior is needed to improve mental health support services in the community. The Patient Activation Measure 13 for Mental Health [11] measures mental health self-management attitudes, and a Japanese version is available [12], but not for self-management behavior. It is desirable to be able to measure not only attitudes, but also behaviors, because motivation and cognitive function are often impaired by mental illness.

The Mental Health Self-management Questionnaire (MHSQ) [13] was developed to measure the use of self-management strategies to recover from mood and anxiety disorders as users empower themselves and take responsibility in their recovery [13]. The items were extracted from qualitative research that clarified strategies to recover from mood and anxiety disorders [14]. MHSQ contains 18 items and consists of three factors (Clinical, Empowerment, and Vitality). Responses are given on a 5-point Likert scale ranging from 0 (Never used) to 4 (Very often used). A higher score indicates more frequent use of self-management strategies over the past two months. The three factors were described as follows: “Clinical” refers to getting help and using resources, “Empowerment” relates to building upon strengths and a positive self-concept to gain control, and “Vitality” refers to an active and healthy lifestyle [13]. MHSQ is reported to have satisfactory reliability, examined by Cronbach’s α (Clinical = .69, Empowerment = .81, Vitality = .75) and the test-retest reliability of each factor using intraclass correlation coefficient (ICC) (Clinical: ICC = .78, 95%CI [.68, .85]; Empowerment: ICC = .76, 95%CI [.65, .84]; Vitality: ICC = .85, 95%CI [.78, .90]) [13]. The validity of the original MHSQ was examined by content validity, concurrent validity, convergent validity, and discriminant validity from the recovery concept [13]. MHSQ is an adequate scale to measure the use of self-management strategy which tends to be highly individualized. Although the original MHSQ focuses on persons with mood and anxiety disorders, it is reasonable to adopt it for other mental diagnosis because of the following points. In the development process of MHSQ, disease-specific items were excluded [13]. It is suggested that the core aspects of self-management for a chronic illness are common, and are as follows: problem solving, decision making, resource utilization, forming a patient/healthcare provider partnership, and taking action [1, 15]. It can be considered that the remaining items were a common strategy to deal with mental health challenges. That is why we recognized that MHSQ can be adopted to other mental diagnoses. Accordingly, it is assumed that if a Japanese version of MHSQ were to be developed, it would contribute to the assessment of mental health self-management behavior in people with mental health challenges.

The aim of this study is to develop a Japanese version of MHSQ (MHSQ-J) and to clarify its psychometric properties among people with mental illness living in Japan. To verify reliability and validity, internal consistency, test-retest reliability, structural validity, hypotheses testing and cross-cultural validity, these components are examined according to the COnsensus-based Standards for the selection of health Measurement Instruments (COSMIN) checklist [16, 17]. We aimed to verify the following hypotheses. MHSQ-J has a positive correlation with scores of the scales associated with self-management behavior and personal recovery and has a negative correlation with scores of the scales associated with clinical recovery.

## Method

### Ethical considerations

The study protocol was approved by the Ethical Committee of the Graduate School of Medicine/Faculty of Medicine, the University of Tokyo (11513). Aims, procedures, the voluntary nature of participation, anonymity, and privacy protection were explained using a Participant Information Sheet. In addition, participants were informed that refusal or suspension of participation would not cause any disadvantage. Participants gave their consent by responding to the questionnaire.

### Study design and participants

This is a validation study for MHSQ-J, and its psychometric properties were validated according to the COSMIN checklist. An anonymous survey administered to people living in the community with mental illness was conducted from July to October in 2017. We recruited 295 outpatients from outpatient mental health clinic A (site A), B (site B), and the psychiatric mental health outpatient service C (site C) in a psychiatric hospital. All the facilities were in the Kanto region. Clinic A is in a commuter town, and clinic B is in an office building of a large city. Site C has over 600 beds, and is separate from an alcohol use disorder specialty outpatient facility.

Inclusion criteria were: outpatients ≥20 years old that understood Japanese. Outpatients who were regarded as having mental health instability by the professional staff working at the site were excluded. Mental health instability was considered in cases of the following: 1) Patients for whom answering this questionnaire would be an excessive burden and participation would be a hindrance to their treatment because of their symptoms or participation in a clinical trial; 2) the relationship with their doctor was not well established because it was a first doctor visit or had just started recently, or, being prone to problems from the past. At site A, as a first step, the doctor asked an eligible patient to participate in this study and to meet the researcher after the consultation. Subsequently, the researcher provided an explanation about this research to patients that agreed to meet us, and obtained consent for participation. At sites B and C, the researcher confirmed patient eligibility or the conditions which the specialists at the facility considered patients to be ineligible. The researcher asked patients about eligibility conditions (e.g., “Is this your first visit to this clinic?”) when necessary. If the patient was eligible, the researcher asked if they would agree to participate in this study. The researcher or facility staff explained the study to patients using a Participant Information Sheet.

Test-retest reliability was tested among participants who agreed to receive the retest by mail (*n* = 104).

### Measurements

#### Development procedure of the Japanese version of MHSQ (MHSQ-J)

The following procedure was used to translate MHSQ, based on the principles described by Wild [18].

1. Preparation and forward translation: first, permission to translate MHSQ into Japanese was obtained from its original author. MHSQ was originally developed in French, and the original author provided an English version for Japanese development. Two native Japanese speakers, who were mental health researchers having experience in psychiatric nursing, carried out independent translations of MHSQ from English to Japanese.
2. Reconciliation: five mental health researchers in face-to-face meetings including the author reached a consensus on a draft Japanese translation of MHSQ that best reflected the literal and conceptual content of the English version of MHSQ.
3. Back translation: the draft version was back-translated into English by a professional native English translator, who did not know about the English version of MHSQ.
4. Back translation review and harmonization: the researchers who conducted the forward translations reviewed the back-translation to ensure the literal and conceptual equivalence of the translation. The original author also confirmed the back translation.
5. Cognitive debriefing and finalization: ten Japanese people using community mental health services tested the pre-final Japanese version of MHSQ to confirm whether the items on the questionnaire were subjectively relevant and appropriate to the situation of their self-management behavior. They were also asked what they thought about the items after answering the questionnaire. The authors confirmed the cognitive equivalence of the translated MHSQ-J (Additional file 1).

#### Scales for verifying hypotheses testing

Five scales were used for hypotheses testing to verify the construct validity of MHSQ-J. The scales assessing correlation with self-management behavior were the Self-management Skill Scale (SMS) [19] and the University of Tokyo Health Sociology version of the SOC scale (SOC-3-UTHS) [20–22]. The scales assessing correlation with recovery were the Japanese version of the Self-identified Stage of Recovery Part-B (SISR-B) [23, 24], the Japanese version of the Flourishing Scale (FS-J) [25, 26], and the Japanese version of the 12-item self-administered version of the WHO Disability Assessment Scale 2.0 (WHODAS 2.0) [27–29].

1. Scales related to Self-management behavior

#### Self-management skill scale

Engaging in desirable health behavior is a part of self-management behavior. Cognitive skills such as effective ways of thinking to achieve the behavior are related to health behavior [30]. The Self-management Skill Scale (SMS) is a scale for measuring general cognitive skills that are effective in realizing the behavior that one wishes and that can be utilized in various situations [19, 31]. The relation of the high score of SMS to various health behaviors was previously confirmed [32, 33]. SMS is a 10-item scale including six reverse-scored items. The items are rated on 4-point Likert scales ranging from 1 (Not applicable) to 4 (Applicable). A higher score indicates an abundance of self-management skill. Cronbach’s α coefficient was reported as .75 among university students in Japan [19].

#### The University of Tokyo health sociology version of the SOC scale ver.1.2

The University of Tokyo Health Sociology version of the SOC scale (SOC-3-UTHS) ver.1.2 is a scale assessing Sense of Coherence (SOC). SOC is a concept that reflects the ability to cope with stress in Salutogenesis theory [34, 35]. As problems in mental health are linked with stress, self-management and the ability to cope with stress was assumed to have a relationship. Positive and significant associations were reported between SOC scores and self-management behavior among people with chronic illness [36]. SOC-3-UTHS ver.1.2 consists of three items. Responses are on a 7-point scale, from 1 (Not applicable) to 7 (Applicable). The items are scored so that a higher score indicates a higher ability to cope with stress. Cronbach’s α coefficient was reported as .84 among a Japanese population [21].

(2) Scales related to recovery

#### Japanese version of self-identified stage of recovery part-B

Personal recovery, an important aspect of recovery from mental illness [9], was assessed using the Japanese version of Self-identified Stage of Recovery Part-B (SISR-B), a 4-item scale to assess the key component of the process of recovery from mental illness [23]. Items are rated on a 6-point scale, ranging from 1 (Disagree strongly) to 6 (Agree strongly). Cronbach’s α coefficient was reported as .82 among people with mental illness living in the community. A higher score indicates a higher level of recovery [24].

#### Japanese version of flourishing scale

The Japanese version of the Flourishing Scale (FS-J) was used to measure psychological well-being as a state of increased personal recovery [37]. FS-J is an 8-item scale. Items are rated on a 7-point scale, ranging from 1 (Strongly disagree) to 7 (Strongly agree). Higher scores indicate respondents that view themselves in positive terms in diverse areas of human functioning. Cronbach’s α coefficient was reported as .95 among undergraduate students in Japan [26].

#### Japanese version of the 12-item self-administered version of WHO disability assessment scale 2.0

Because self-management affects clinical recovery, such as through a reduction of symptoms or disability [9], the Japanese version of the 12-item self-administered version of the WHO Disability Assessment Scale 2.0 (WHODAS 2.0) was used to assess the level of clinical recovery. This scale assesses the degree of disability due to various health conditions, with responses on a 5-point scale ranging from 1 (None) to 5 (Extreme/cannot do), where a higher score indicates a higher level of disability [29]. Cronbach’s α coefficient was reported as .92 among preoperative patients in Japan [38].

#### Demographic variables

Information regarding socio-demographic and clinical characteristics was collected, including age, sex, income, marital status, work status, educational background, cohabitants, diagnoses of psychiatric disorders, period from first visit to psychiatrist, an experience of hospitalization due to mental health problems, the use of mental health support services, frequency of visits with psychiatrists, and severities of depression and anxiety. The severities of depression and anxiety were assessed using the Japanese version of the Patient Health Questionnaire-9 (PHQ-9) [39–41] and the Japanese version of General Anxiety Disorder-7 (GAD-7) [41, 42], respectively. PHQ-9 has nine items, and GAD-7 has seven items. Both are based on 4-point scales. Cronbach’s α coefficient of PHQ-9 was reported as .91 among university students [43], and that of GAD-7 was reported as. 87 among university students in Japan [44].

### Statistical analysis

Statistical analysis was conducted for respondents who completely answered all items of MHSQ-J.

#### Validity of MHSQ-J

Since there was no gold standard for assessing self-management strategy, validity was determined by assessing construct validity. Construct validity was confirmed by structural validity, cross-cultural validity, and hypotheses testing.

According to the COSMIN checklist [16], confirmatory factor analysis (CFA) was performed to confirm the fit of the factor structure of the original MHSQ to the data for structural validity. Cross-cultural validity was also examined by CFA. The following indices were used: Adjusted Goodness of Fit Index (AGFI), Comparative Fit Index (CFI), and Root Mean-Square Error of Approximation (RMSEA). AGFI and CFI values equal to or above .95 were considered a good fit [45, 46]. An RMSEA value less than or equal to .06 was considered a good fit, .08 or less indicates reasonable fit, .08 to .10 indicates a mediocre fit, and values above .10 indicate a poor fit [46, 47]. Factorial correlations were also examined.

Exploratory factor analysis (EFA) was performed to verify structural validity along with CFA. The Kaiser-Meyer-Olkin index (KMO) of sampling adequacy and Bartlett’s chi-square test of sphericity with *p* < .05 were examined to confirm the suitability of the data for factor analysis. KMO was compared to adequacy of standards [48]. The generalized least-squares method with Promax rotation was used for the factor extraction because it was assumed that the factors have correlations with each other. The number of factors was determined based on the Scree test.

Pearson’s correlation coefficients were examined between the scales for hypotheses testing and the total score and each factor of MHSQ-J. The hypotheses for MHSQ-J score were as follows: positive associations with the scores of SMS, SOC-UTHS ver.1.2, SISR-B, and FS-J, moderate negative association was assumed with WHODAS 2.0. Pearson’s correlation coefficients of .40–.70 and > .70 were considered moderate and strong, while .20–.40 and < .20 were considered weak and poor correlations, respectively.

#### Reliability of MHSQ-J

To evaluate reliability, internal consistency and test-retest reliability were calculated. Cronbach’s α coefficient for total score and each subscale of MHSQ-J were examined to assess internal consistency. Sufficient internal consistency was assumed for a Cronbach’s α greater than .70 [49, 50]. Test-retest reliability was tested among participants who agreed to receive the retest by mail (*n* = 104). Participants were asked to take a re-administration of MHSQ-J two weeks later after the initial questionnaire and we used data from all responses (*n* = 82) that were provided within a period of 8–20 days from the initial survey. Test-retest reliability was evaluated by exclusion of responses that reported significant changes in their condition between the initial survey and the test-retest survey. Participants were also asked about changes in their subjective condition (“Were there any changes in your condition since the time you previously answered this survey?”) using an original item on a 7-point Likert scale ranging from “7: Extremely improved” to “1: Extremely worsened” to confirm significant differences in their condition compared with the initial survey. We judged that there was a significant change in condition at the retest if the participants answered the question as 1, 2, 6, or 7. For test-retest reliability, ICC was examined for the total score and each subscale of MHSQ-J using the data with complete answers for MHSQ-J. ICC > .70 considered as excellent agreement [51].

Statistical analyses, except for CFA, were conducted using SPSS 22.0 for Windows. CFA was conducted using AMOS version 22. Values of p less than .05 were considered statistically significant (two-tailed test).

## Results

### Respondent characteristics and scale descriptions

A total of 266 participants returned the questionnaire (response rate = 90%) and 82 returned the retest (response rate = 79%). For all analyses, 23 respondents were excluded because of incomplete answers in MHSQ-J, and a total of 243 were used. Socio-demographic and clinical characteristics of the respondents are shown in Table 1.

**Table 1:** Socio-demographic and clinical characteristics of the respondents

	Total population *N* = 243	
Characteristics	n (Mean)	% [SD]
Sex		
Male	128	52.7
Missing	4	1.6
Age		
	(46.3)	[12.3]
Missing	9	3.7
Marital status		
Not married	137	56.4
Married	65	26.7
Divorced/Widowed	36	14.8
Other	1	0.4
Missing	4	1.6
Income (Yen)		
< 2,500,000	52	21.4
< 4,500,000	54	22.2
< 7,000,000	31	12.8
≥ 7,000,000	26	10.7
Livelihood protection	38	15.6
Unknown, Answer refused	34	14
Missing	8	3.3
Educational background		
Junior high school	17	7.0
High school	59	24.3
Junior college /vocational school	49	20.2
Bachelor’s degree or higher	110	45.3
Missing	8	3.3
Work status		
Working		
Regular employment	53	21.8
Part time job	24	9.9
Others	42	17.3
Not working	110	45.3
Missing	14	5.8
Diagnosis^a^		
Depression	100	41.2
Bipolar Disorder	32	13.2
Anxiety disorder	43	17.7
Substance use disorder	9	3.7
Developmental disorder	34	14.2
Schizophrenia	72	29.6
Unknown	5	2.1
Others	27	11.1
Missing	4	1.6
Use of support^a^		
Visiting nurse	34	14.0
Psychiatric day care / day night care	24	9.9
Employment support service	28	11.5
Return to work program	9	3.7
Psychotherapy	12	4.9
Other	52	21.4
Nothing particular	128	52.7
Missing	25	10.3
PHQ-9		
0–9 (Minimal to Mild)	122	50.2
10–14 (Moderate)	62	25.5
15–27 (Moderately severe to Severe)	55	22.6
Missing	4	1.6
GAD-7		
0–9 (Minimal to Mild)	171	70.4
10–14 (Moderate)	43	17.7
15–21 (Severe)	26	10.7
Missing	3	1.2

^a^: Multiple answers

*PHQ-9* Patient Health Questionnaire-9, *GAD-7* General Anxiety Disorder-7

The mean age was 46.3 (SD = 12.3), and the range was between 21 and 82. Almost half of the respondents were male and not married. There were 44% who lived alone, and 41% had hospitalization experience because of mental problems. The mean frequency of hospital/clinic visits per month was 2.3 (SD = 2.0). Average period from the first visit to the psychiatric/psychosomatic doctor was 11.9 years (SD = 9.8). The mean score of total MHSQ was 38.5 (SD =11.5, range 5–71). A ceiling effect was seen with item 5, and a floor effect was seen with items 4 and 18.

### Validity of MHSQ-J

#### Structural and cross-cultural validity

**Fig. 1:**
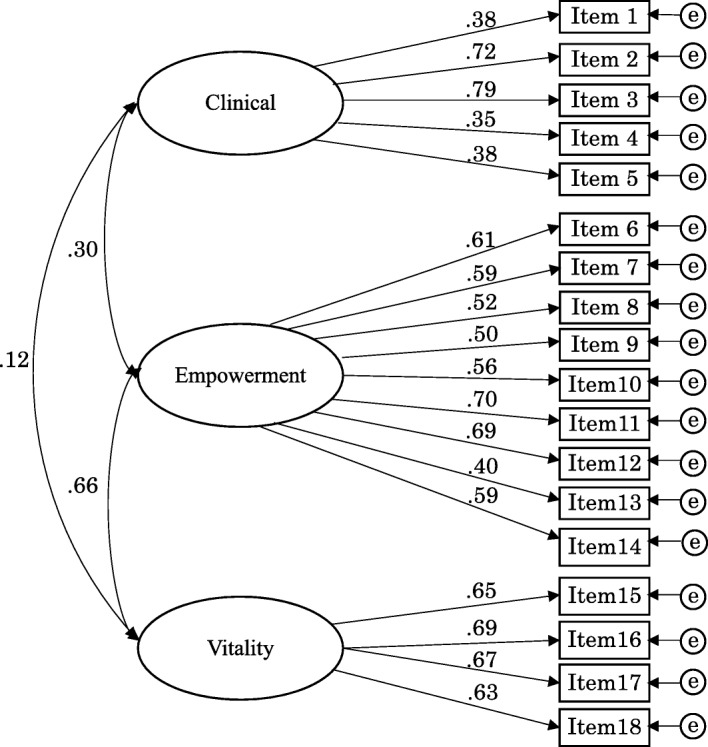
Confirmatory factor analysis of MHSQ-J

CFA was performed with all items, based on the structure of the original MHSQ (Fig. 1: Confirmatory factor analysis of MHSQ-J). The goodness-of-fit indices were not optimal, but marginal (AGFI = .830, CFI = .852, RMSEA = .072). The factorial correlation between Empowerment and Vitality was .66. The correlations between Clinical and the other two factors were .30 with Empowerment, and .15 with Vitality.

The adequacy of data for EFA was confirmed by Bartlett’s test of sphericity (*p* < .001) and KMO (.842). The goodness-of-fit test for the factor extraction method was adequate (χ^2^ = 120.381, df = 102, *p* = .103). The three-factor-structure indicated by the Scree plot was optimal. The three factors of MHSQ-J explained 47.83% of the variance. Whole factor loadings are shown in Table 2. Three items (item 4, 13, and 14) loaded onto two factors with low factor loadings (< .40) and had comparable values over .20. The inspection of kurtosis and skewness indices for the total score and each factor were tested, and the results were: Total: kurtosis −.382, skewness −.018; Clinical: kurtosis −.224, skewness −.033; Empowerment: kurtosis −.347, skewness −.189; Vitality: kurtosis −.596, skewness −.042.

**Table 2:** Means, standard deviations (SDs), factor loadings, and Cronbach’s α coefficients for the total score and MHSQ-J factors (*N* = 243)

Items of MHSQ-J^a^ (α = .83) and original factors with Cronbach's α coefficient	Mean	SD	Factor loading^b^			Cronbach's α coefficient if the item was deleted	
			Factor 1	Factor 2	Factor 3	For the total	For the original factor
**Clinical factor (α = .65)**	10.4	3.9					
1 I look for available resources to help me with my difficulties (websites, organizations, healthcare professionals, books, etc.).	1.7	1.3	.096	.032	.363	.83	.63
2 I consult with a professional (a physician, psychologist, social worker, etc.) for my mental health problem.	2.2	1.2	-.039	.033	.745	.83	.54
3 I get actively involved in my follow-up with the healthcare professionals I consult (physician, psychologist, social worker, etc.).	2.1	1.4	.028	.009	.722	.83	.51
4 I participate in a support or help group in order to help me manage the difficulties I’m experiencing.	0.8	1.2	-.075	.222	.347	.83	.64
5 I take medication for my mental health problem, following the indications of a healthcare professional.	3.6	0.9	.110	-.047	.379	.83	.65
**Empowerment factor (α = .81)**	20.6	7.3					
6 I try to solve my difficulties one step at a time.	2.5	1.1	.546	.032	.204	.82	.79
7 I try to recognize the warning signs of a relapse of my mental health disorder.	2.4	1.3	.524	.011	.300	.82	.79
8 I learn to differentiate between my mental health problem and myself as a person.	1.7	1.4	.613	-.164	.162	.82	.80
9 I focus my attention on the present moment.	2.2	1.2	.564	-.107	.091	.82	.80
10 I learn to live with my strengths and weaknesses.	2.6	1.2	.582	-.041	.018	.82	.80
11 I congratulate myself on my successes, whether small or large.	2.1	1.2	.718	.124	-.188	.82	.78
12 I try to love myself as I am.	1.8	1.3	.690	.218	-.299	.82	.79
13 I take my capabilities into account when arranging my schedule.	2.4	1.2	.231	.246	-.019	.83	.82
14 I find comfort and an attentive ear in the people around me.	2.3	1.3	.262	.386	.165	.82	.80
**Vitality factor (α = .75)**	8.3	3.9					
15 I engage in activities I like in order to maintain an active life.	2.6	1.2	.080	.578	.083	.82	.71
16 I engage in sports, physical activity.	1.9	1.3	-.229	.870	-.031	.83	.66
17 I have a healthy diet.	2.4	1.3	.090	.599	-.027	.82	.70
18 I do exercises to relax (yoga, tai-chi, breathing techniques, etc.).	1.4	1.4	-.034	.670	.110	.82	.71

a: The latest English version (2017) of MHSQ was obtained from the author of MHSQ by private communication

b: Factor loadings were based on exploratory factor analysis with generalized least-squares method and promax rotation

#### Hypotheses testing

Significant correlations except for the Clinical subscale were confirmed as being in the same direction as the hypotheses (Table 3). The total score, the Empowerment subscale and the Vitality subscale of MHSQ-J showed a significant positive correlation with SISR-B, FS-J, SOC-3-UTHS ver.1.2 and SMS, and a significant negative correlation with WHODAS 2.0. The Clinical subscale showed a significant correlation only with WHODAS 2.0, and the correlation was very weak.

**Table 3:** Pearson’s Correlation Coefficients between the total and each MHSQ-J factor with the score of hypotheses testing measures

	n	Pearson’s correlation coefficients				Cronbach’s α coefficients
		MHSQ-J total	Clinical factor	Empowerment factor	Vitality factor	
SMS	236	.321^b^	−.066	.365^b^	.325^b^	.77
SOC-3-UTHS ver. 1.2	237	.341^b^	−.050	.361^b^	.371^b^	.81
SISR-B	236	.437^b^	−.010	.519^b^	.410^b^	.85
FS-J	235	.538^b^	.019	.578^b^	.467^b^	.85
WHODAS 2.0	217	−.296^b^	.157^a^	−.347^b^	−.385^b^	.86

^a^ < .05, ^b^ < .001

*SISR-B* Self-identified stage of recovery -B, *FS-J* Japanese version of the Flourishing Scale, *SOC-3-UTHS* The University of Tokyo Health Sociology version of the SOC scale, *SMS* Self-management Skill Scale, *WHODAS* WHO Disability Assessment Schedule

#### Reliability of MHSQ-J

1. Internal consistency

The Cronbach’s α coefficients of the total score and each subscale of MHSQ-J, and the coefficient when an item was deleted, are shown in Table 2. Cronbach’s α of the total score, the Clinical subscale, the Empowerment subscale, and the Vitality subscale were .83, .65, .81, and .75, respectively.

(2) Test-retest reliability

In total, there were 82 responses for the retest, and 20 responses were excluded because of the following reasons: incomplete answers for MHSQ-J (*n* = 7), significant change in condition at the retest (*n* = 9), not within the period of 8–20 days from the initial survey (*n* = 3), or the reply date of the retest was unidentified (*n* = 1). Finally, 62 responses were used to examine test-retest validity, and the mean period of the two tests was 13.9 days (SD = 1.83, range = 11–20).

The ICC scores of MHSQ-J were as follows: Total score: .84, 95% confidence interval (CI) [.75, .90], Clinical: .75, 95% CI [. 62, .84], Empowerment: .81, 95% CI [.70, .88], and Vitality: .62, 95% CI [.44, .75]. Overall, the scores showed moderately good test-retest reliability. The ICC score of each item of the Vitality subscale were also examined. The scores were as follows, item 15: .32, 95% CI [.08, .53], item 16: .66, 95%CI [.50, .78], item 17: .58, 95% CI [.39, .73], and item 18: .65, 95% CI [.47, .77].

## Discussion

This study aimed to develop and verify the reliability and validity of MHSQ-J. The results indicated adequate internal consistency, test-retest reliability, structural validity, and cross-cultural validity.

Regarding structural validity, the result for the CFA using the original factorial structure showed almost acceptable factor loadings and a marginal goodness-of-fit index score. The result for the EFA was that the highest factor loadings of items 13 and 14 were for Vitality, although these items of the original MHSQ loaded in Empowerment. But it is reasonable to suppose that they belong to Empowerment as on the original scale, based on the size of the factor loadings. Therefore, it seems reasonable to consider the structure of MHSQ-J as similar to the original MHSQ among the participants in this study. The correlations between factors were moderate and positive between the Empowerment factor and the Vitality factor, but weak between the Clinical factor and the other two factors. This trend was similar to the original results (r = .48 between Empowerment and Vitality, r = .23 between Clinical and Empowerment, and r = .28 between Clinical and Vitality), and the original author indicated that the Empowerment and the Vitality subscales of MHSQ might contain related sub-aspects which differ from the Clinical subscale [13].

Concerning hypotheses testing, the scale related to self-management and recovery showed significant correlations with the total score, the Empowerment subscale, and the Vitality subscale of MHSQ-J in the hypothesized direction. On the other hand, the Clinical subscale showed no correlation with any scales used for hypotheses testing in this study. This seems consistent with the results for the Clinical subscales of the original MHSQ, which showed a significant correlation of smaller than .30 on the scales of symptoms and on the social participation scale [13]. The original author explained that “strategies from the Clinical subscale, such as consulting a mental health professional, might be implemented earlier in the recovery process when symptoms are most severe” [13]. Furthermore, while WHODAS 2.0 was used to assess mental health clinical recovery, it might not be sensitive enough to measure clinical recovery in mental illness. WHODAS 2.0 measures the disability caused from all health problems including physical problems, which might be one of the reasons weakening the correlation between the Clinical subscale and clinical recovery.

In this study, MHSQ-J showed good reliability in terms of reasonable Cronbach’s α coefficients and ICCs. The Cronbach’s α coefficients indicated excellent internal consistency in the total score, the Empowerment subscale, and the Validity subscale. The internal consistency of the Clinical subscale was acceptable, but relatively low. This result was consistent with the results for the original MHSQ (Clinical = .69, Empowerment = .81, Vitality = .75) [13].

The ICCs of the total score, the Clinical subscale, and the Empowerment subscale were satisfactory, and the ICC of the Vitality subscale was acceptable, but relatively low, especially for item 15.

In addition, a ceiling effect on item 5 (the use of medicine), and a floor effect on items 4 and 18 (participation in a group which supports or helps oneself, and exercises to relax, respectively), were seen in this study. Regarding the ceiling effect, most of the participants took medication frequently, because they were all outpatient users. Regarding the floor effect, the groups described in item 4 and the activity for relaxation described in item 18 are not familiar to all people in Japan with mental illness.

This study has limited generalizability. Participants were limited to psychiatric outpatients. And the participants were also limited in terms of who could answer the questionnaire and their symptoms, such that cognitive function and concentration seemed to be high. We did not collect information about the diagnosis from the clinic. This was also a limitation of this study as we could not eliminate a participant that uses psychiatric or psychosomatic outpatient services without psychiatric illness.

This is the first scale to measure the usage of mental health self-management strategies in Japan. In this study, the small difference between the original and the Japanese version of MHSQ in model fit and factor structure probably is based on cross-cultural differences and differences in the participants. Although we developed and analyzed this scale faithfully to the original scale, the results indicate the need to modify some items from a cross-cultural and trans-diagnostic point of view. The present study could not clarify the nature of each factor in MHSQ-J. To provide greater usefulness for MHSQ-J, and an understanding of self-management for mental health, further study is needed to more readily adapt services for people with mental illness living in the community in Japan, and to clarify which factors are more important at various points of treatment, or depending on symptoms.

## Conclusion

MHSQ-J is valid and it reliably measures the use of self-management strategies for mental health among people with mental illness living in a Japanese community.

## Additional file

Additional file 1: Japanese version of Mental Health Self-management Questionnaire (MHSQ-J). (DOCX 33 kb)

## Abbreviations

AGFI: Adjusted Goodness of Fit Index
CFA: confirmatory factor analysis
CFI: Comparative fit index
COSMIN: COnsensus-based Standards for the selection of health Measurement Instruments
EFA: Exploratory factor analysis
FS-J: Japanese version of the Flourishing Scale
GAD-7: Japanese version of General Anxiety Disorder-7
ICC: Intraclass correlation coefficient
KMO: Kaiser-Meyer-Olkin index
MHSQ: Mental health self-management questionnaire
MHSQ-J: Japanese version of mental health self-management questionnaire
PHQ-9: Japanese version of patient health questionnaire-9
QOL: Quality of Life
RMSEA: Root Mean-Square Error of Approximation
SISR-B: Self-identified Stage of Recovery Part-B
SMS: Self-management Skill Scale
SOC: Sense of Coherence
SOC-3-UTHS: The University of Tokyo Health Sociology version of the SOC scale
WHODAS 2.0: WHO Disability Assessment Scale 2.0

## References

[1] Lorig KR, Holman H (2003). Self-management education: history, definition, outcomes, and mechanisms. Ann Behav Med.

[2] Foster G, Taylor SJC, Eldridge SE, Ramsay J, Griffiths CJ. Self-management education programmes by lay leaders for people with chronic conditions. Cochrane Database Syst Rev. 2007;(4):CD005108. 10.1002/14651858.CD005108.pub2 17943839

[3] Newman S, Steed L, Mulligan K (2004). Self-management interventions for chronic illness. Lancet.

[4] National Institute for Health and Care Excellence. Bipolar disorder: assessment and management 2014(NICE Clinical guideline 185). 2014. https://www.nice.org.uk/guidance/cg185. Accessed 6 June 2017. 31487127

[5] Lam RW, McIntosh D, Wang J, Enns MW, Kolivakis T, Michalak EE, Sareen J, Song W-Y, Kennedy SH, MacQueen GM (2016). Canadian network for mood and anxiety treatments (CANMAT) 2016 clinical guidelines for the management of adults with major depressive disorder: section 1. Disease burden and principles of care. Can J Psychiatry.

[6] Australian Health Ministers’ Advisory Council. A national framework for recovery-oriented mental health services 2013. (2013) http://www.health.gov.au/internet/main/publishing.nsf/content/67d17065514cf8e8ca257c1d00017a90/$file/recovgde.pdf. Accessed 7 June 2017.

[7] van Schie D, Castelein S, van der Bijl J, Meijburg R, van Stringer B, van Meijel B (2016). Systematic review of self-management in patients with schizophrenia: psychometric assessment of tools, levels of self-management and associated factors. J Adv Nurs.

[8] Houle J, Gascon-Depatie M, Bélanger-Dumontier G, Cardinal C (2013). Depression self-management support: a systematic review. Patient Educ Couns.

[9] Anthony WA. Recovery from mental illness: the guiding vision of the mental health service system in the 1990s. Psychosoc Rehabil J. 1993;16(4):11–23.

[10] Provencher HL, Keyes CLM (2011). Complete mental health recovery: bridging mental illness with positive mental health. J Public Mental Health.

[11] Hibbard JH, Stockard J, Mahoney ER, Tusler M (2004). Development of the patient activation measure (PAM): conceptualizing and measuring activation in patients and consumers. Health Serv Res.

[12] Fujita E, Kuno E, Kato D, Kokochi M, Uehara K (2010). Development and validation of the Japanese version of the patient activation measure 13 for mental health. Clin Psychiatry.

[13] Coulombe S, Radziszewski S, Trépanier SG, Provencher H, Roberge P, Hudon C, Meunier S, Provencher MD, Houle J (2015). Mental health self-management questionnaire: development and psychometric properties. J Affect Disord.

[14] Villaggi B, Provencher H, Coulombe S, Meunier S, Radziszewski S, Hudon C, Roberge P, Provencher MD, Houle J. Self-Management Strategies in Recovery From Mood and Anxiety Disorders. Global Qualitative Nursing Research 2015;2:2333393615606092. 10.1177/2333393615606092 PMC5342854 28462317

[15] Lorig K (2006). Living a healthy life with chronic conditions: self-management of heart disease, arthritis, stroke, diabetes, asthma, bronchitis, emphysema and others.

[16] Mokkink LB, Terwee CB, Patrick DL, Alonso J, Stratford PW, Knol DL, Bouter LM, de Vet HC (2012). COSMIN checklist manual.

[17] Terwee CB, Mokkink LB, Knol DL, Ostelo RW, Bouter LM, de Vet HC (2012). Rating the methodological quality in systematic reviews of studies on measurement properties: a scoring system for the COSMIN checklist. Qual Life Res.

[18] Wild D, Grove A, Martin M, Eremenco S, McElroy S, Verjee-Lorenz A, Erikson P (2005). Principles of good practice for the translation and cultural adaptation process for patient-reported outcomes (PRO) measures: report of the ISPOR task force for translation and cultural adaptation. Value Health.

[19] Takahashi H, Nakamura M, Kinoshita T, Masui S (2000). Development and validation of a self-management skill scale. Jpn J Public Health.

[20] Antonovsky A (1987). Unraveling the mystery of health: how people manage stress and stay well.

[21] Togari T, Yamazaki Y, Nakayama K, Shimizu J (2007). Development of a short version of the sense of coherence scale for population survey. J Epidemiol Community Health.

[22] Togari T. 3 koumoku-ban-SOC-shakud (SOC3-UTHS ver1.2) ni-tsuite. http://d.hatena.ne.jp/ttogari-tky/files/3%E9%A0%85%E7%9B%AE%E7%89%88SOC%E5%B0%BA%E5%BA%A6.pdf. Accessed 6 July 2017. (In Japanese).

[23] Andresen R, Oades L, Caputi P (2003). The experience of recovery from schizophrenia: towards an empirically validated stage model. Aust N Z J Psychiatry.

[24] Chiba R, Kawakami N, Miyamoto Y, Andresen R (2010). Reliability and validity of the Japanese version of the self-identified stage of recovery for people with long term mental illness. Int J Ment Health Nurs.

[25] Diener E, Wirtz D, Tov W, Kim-Prieto C, Choi D, Oishi S, Biswas-Diener R (2010). New well-being measures: short scales to assess flourishing and positive and negative feelings. Soc Indic Res.

[26] Sumi K (2014). Reliability and validity of Japanese versions of the flourishing scale and the scale of positive and negative experience. Soc Indic Res.

[27] Tazaki M, Yamaguchi T, Yatsunami M, Nakane Y (2014). Measuring functional health among the elderly: development of the Japanese version of the World Health Organization disability assessment schedule II. Int J Rehabil Res.

[28] Ustun TB, K N, Chatterji S, Rehm J (2010). Measuring health and disability: manual for WHO disability assessment schedule (WHODAS 2.0).

[29] World Health Organisation. Disability assessment schedule. WHO Disability Assessment Schedule 2.0. http://www.who.int/classifications/icf/whodasii/en/. Accessed 6 July 2017.

[30] Jingu H (1993). Sukiru-no-ninchi-shinrigaku-koudou-no-puroguramu-wo-kangaeru.

[31] Sakuma H, Takahashi H, Takehana Y, Hisano Y (2009). The relationship between high school student’s stress responses and self-management skills. Jpn J School Health.

[32] Takehana Y, Takahashi H (2002). Relationship between self-management behavior and cognitive skills in type 2 diabetes mellitus patients. Jpn J Public Health.

[33] Fujiyoshi M, Tsutsui A, Matsuoka N, Hanioka T (2005). Analyses in the factors of toothbrushing behavior and knowledge, and attitude toward toothbrushing, and gingivitis and plaque accumulation status. J Dental Health.

[34] Antonovsky A (1993). The structure and properties of the sense of coherence scale. Soc Sci Med.

[35] Antonovsky A (1996). The salutogenic model as a theory to guide health promotion. Health Promot Int.

[36] Aujoulat I, Mustin L, Martin F, Pélicand J, Robinson J. The application of Salutogenesis to health development in youth with chronic conditions. In: The handbook of Salutogenesis. edn.: Springer; 2017: 337–344. 28590662

[37] Slade M (2010). Mental illness and well-being: the central importance of positive psychology and recovery approaches. BMC Health Serv Res.

[38] Ida M, Naito Y, Tanaka Y, Matsunari Y, Inoue S, Kawaguchi M (2017). Feasibility, reliability, and validity of the Japanese version of the 12-item World Health Organization disability assessment Schedule-2 in preoperative patients. J Anesth.

[39] Kroenke K, Spitzer RL, Williams JB (2001). The phq-9. J Gen Intern Med.

[40] Muramatsu K, Kamijima K, Yoshida M, Otsubo T, Miyaoka H, Muramatsu Y, Gejyo F (2007). The patient health questionnaire, Japanese version: validity according to the mini-international neuropsychiatric interview–plus. Psychol Rep.

[41] Muramatsu K (2014). An up-to-date letter in the Japanese version of PHQ, PHQ-9, PHQ-15. Niigata Seiryou--daigaku-daigakuin-rinshou-sinrigaku-kenkyu.

[42] Spitzer RL, Kroenke K, Williams JB, Löwe B (2006). A brief measure for assessing generalized anxiety disorder: the GAD-7. Arch Intern Med.

[43] Umegaki Y, Todo N (2017). Psychometric properties of the Japanese CES-D, SDS, and PHQ-9 depression scales in university students. Psychol Assess.

[44] Masunaga K, Sugiura Y (2018). Moderating effect of well-being and gratitude on the relationships between negative metacognitive beliefs and generalized anxiety/depressive symptoms.

[45] Hu Lt BPM (1999). Cutoff criteria for fit indexes in covariance structure analysis: conventional criteria versus new alternatives. Struct Equ Model Multidiscip J.

[46] Schreiber JB (2008). Core reporting practices in structural equation modeling. Res Soc Adm Pharm.

[47] Hooper D, Coughlan J, Mullen M. Structural equation modelling: guidelines for determining model fit. Articles. 2008;2.

[48] Kaiser HF (1974). An index of factorial simplicity. Psychometrika.

[49] Cortina JM (1993). What is coefficient alpha? An examination of theory and applications. J Appl Psychol.

[50] Terwee CB, Bot SD, de Boer MR, van der Windt DA, Knol DL, Dekker J, Bouter LM, de Vet HC (2007). Quality criteria were proposed for measurement properties of health status questionnaires. J Clin Epidemiol.

[51] Bartko JJ (1966). The intraclass correlation coefficient as a measure of reliability. Psychol Rep.

