# Supplementary material for: Reliability and validity of the Japanese version of the Mental Health Self-management Questionnaire among people with mental illness living in the community
Source: BMC Psychol. 2019 May 22;7:30. doi: 10.1186/s40359-019-0301-4 (PMC6532246; doi:10.1186/s40359-019-0301-4)
Supplement: Supplementary file 1 — Japanese version of Mental Health Self-management Questionnaire (MHSQ-J). (DOCX 33 kb) [file 40359_2019_301_MOESM1_ESM.docx]

Additional file 1: The Japanese version of Mental Health Self-management Questionnaire (MHSQ-J)

【MHSQ-J】

この質問票は、気分を改善したり、良好なこころの健康状態を維持したり、再発を防ぐために用いられる可能性のある方法についてお尋ねするもので、以下に様々な方法をお示ししています。

それぞれの方法について、この2ヶ月間で、あなたがどのくらいのでそれを用いたか

（0）全くしなかった　から　（4）とてもに　の中からお選びください。

正しい答えや間違った答えがあるものではありません。ありのままお答えください。

|  |  | 全く  しな  かった | とてもまれに | 時々 | たび  たび | とてもに |
| --- | --- | --- | --- | --- | --- | --- |
| １ | 困難を抱える自分自身の助けになる、利用可能な資源を探す （組織や団体、保健医療の専門家、本、インターネット情報など）。 | 0 | １ | ２ | ３ | ４ |
| ２ | 自分のこころの健康上の問題について専門家（医師、心理士、 ソーシャルワーカーなど）に相談する。 | 0 | １ | ２ | ３ | ４ |
| ３ | 自分の相談先の保健医療の専門家（医師、心理士、ソーシャル ワーカーなど）との面談・診察場面に、積極的に参加する。 | 0 | １ | ２ | ３ | ４ |
| ４ | 自分の体験している困難とうまく付き合う助けとなるよう、 サポートグループや自助グループに参加する。 | 0 | １ | ２ | ３ | ４ |
| ５ | 自分のこころの健康上の問題に対して、医療者の指示に沿って 薬を使用する。 | 0 | １ | ２ | ３ | ４ |
| ６ | 自分自身の抱える困難に対して一歩ずつ解決を試みる。 | 0 | １ | ２ | ３ | ４ |
| ７ | こころの不調が再発する前兆を把握するよう試みる。 | 0 | １ | ２ | ３ | ４ |
| ８ | こころの健康上の問題と、一人の人としての自分を区別するよう意識している。 | 0 | １ | ２ | ３ | ４ |
| ９ | 今のこの瞬間に注意を向ける。 | 0 | １ | ２ | ３ | ４ |
| 10 | 自分には強みと弱みがあると意識して生活する。 | 0 | １ | ２ | ３ | ４ |
| 11 | 物事の大小に関わらず、達成できた自分をほめる。 | 0 | １ | ２ | ３ | ４ |
| 12 | そのままの自分を愛するよう努める。 | 0 | １ | ２ | ３ | ４ |
| 13 | 自分の許容量を考えて予定を立てる。 | 0 | １ | ２ | ３ | ４ |
| 14 | 自分のまわりに、安らぎや、話を聞いてくれる人を見つけ出す。 | 0 | １ | ２ | ３ | ４ |
| 15 | 意欲的な生活を維持するために、自分の好きな活動をする。 | 0 | １ | ２ | ３ | ４ |
| 16 | 身体を動かしたり、スポーツをしたりする。 | 0 | １ | ２ | ３ | ４ |
| 17 | 健康的な食事をする。 | 0 | １ | ２ | ３ | ４ |
| 18 | リラックスするための活動（ヨガ、太極拳、呼吸法など）をする。 | 0 | １ | ２ | ３ | ４ |
